# Supplementary material for: Artificial intelligence for the classification of fractures around the knee in adults according to the 2018 AO/OTA classification system
Source: PLoS One. 2021 Apr 1;16(4):e0248809. doi: 10.1371/journal.pone.0248809 (PMC8016258; doi:10.1371/journal.pone.0248809)
Supplement: S1 File — (DOCX) [file pone.0248809.s001.docx]

S1 File

# Data & code

The test data set is available at <https://datasets.aida.medtech4health.se/10.23698/aida/kf2020> and the code used for training the network is available from GitHub at <https://github.com/AliRazavian/TU> (commit 19c29cb3fc2bf5cb44912887ede317947b5a4bf5).

# Further description of AO/OTA output categories

The first two numbers in the AO/OTA classification label [1] represent location; bone and bone segment. The AO-OTA then divides fractures based on morphological features into types, denoted by capital letters A-C. Next, fractures are divided into groups, represented by numbers 1-3 and further into subgroups, also represented by numbers 1-3. Some fracture categories also have additional qualifiers to describe them represented by lowercase letters [1]. (Supplemental figure 1)

**41A1.1(a)**

**bone bone segment type group subgroup qualifier**

***S1 Fig.*** *Example of how label numbers and letters are used*

## Proximal tibia fractures (41)

The proximal tibia is denoted as 41 [1]. Type A denotes extra articular fractures. Avulsion fractures (A1), simple extra articular fractures (A2) and extra-articular wedge and multifragmentary fractures (A3) were grouped in this category. Type B, partially articular fractures, constituted the tibial plateau fractures which were classified as split (B1), depression (B2) and split/depression (B3) [1]. Split and split/depression was for the purpose of this study interpreted as fractures with an oblique fracture line extending from articular to metaphyseal cortex. Further subgrouping was based on laterality where B1.1, B2.1, and B3.1 are lateral, B1.2. B2.2. and B.3.2 medial and B1.3 and B3.3 have an oblique component [1]. Type C encompasses bicondylar plateau fractures as well as other fractures with an articular component and metaphyseal-diaphyseal disassociation [1]. We applied qualifiers to tibia B-type lateral fractures based on where the depression fracture was located. The letter x denotes a depression fracture in the center of the lateral plateau, t denotes anterolateral fractures and u denotes posterolateral fractures. Depression (B2) and split depression (B3) fractures were pooled together for this analysis as both contain a depressed fracture component.

## Patella fractures (34)

The patella is denoted by the number 34 [1]. Avulsion fractures or fissures that did not completely separate the patella in the transverse plane were typed as A. The patella A type has only one group, A.1, the avulsion fracture. It has no further subgroups, instead the following qualifiers are used to denote location of avulsion a - proximal, b - distal, c -lateral and d -proximal [1]. Sagittal/vertical fractures were typed as B and fractures with a transverse main component were typed as C in accordance with the AO-OTA guidelines [1]. Types B and C were grouped and sub-grouped based on comminutions and laterality [1].

## Distal femur fractures (33)

The distal femur is denoted by the number 33 [1]. Epicondylar avulsion fractures (A1) and extra articular simple (A2) wedge (A3) and multi-fragmentary (A3) fractures were typed as A. Partially articular sagittal condylar fractures were typed as B and grouped into lateral (B1) or medial (B2) fractures. Also typed as B, partially articular, were the anterior and lateral flake fractures (B3.1) and the “Hoffa fractures” (B3.2, B3.3). Type C, fractures with fracture lines separating the condyles as well as lines causing metaphyseal-diaphyseal separation were typed here and further grouped based on level of comminution [1].

# Additional results

The dataset contained 153 fractures where the network exhibited an 87% sensitivity and 86% specificity with a Youden’s J being 0.73. AUC was estimated to be 0.90 (95% CI 0.86 to 0.93). Out of the four with lowest probability of fractures, two were patella fractures, one was a hip fracture and one had an external fixator that most likely interfered, see below figure.


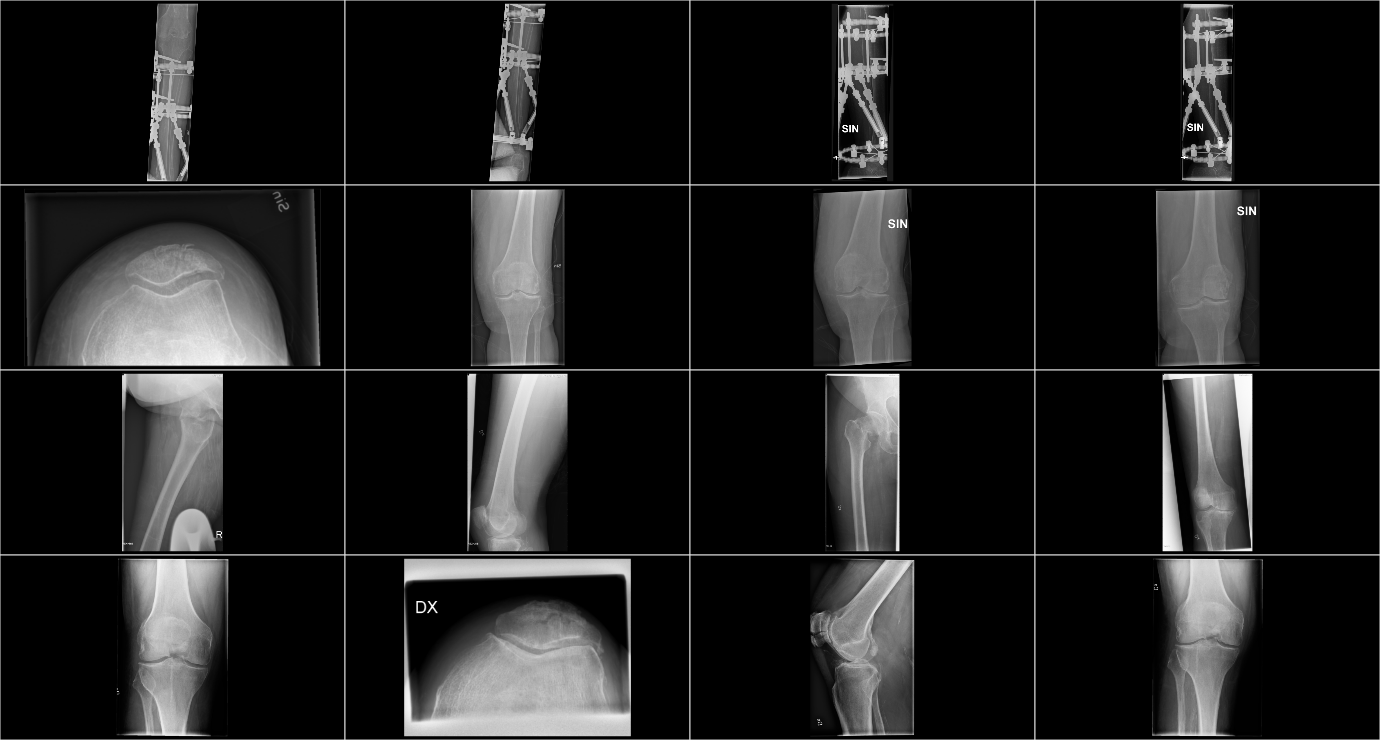


# Classification base data

Distribution of AO/OTA classes in the training data as well as test set. The “maybe” outcome was treated as “no fracture” during testing and training. Besides the outcome categories that the network was evaluated for in this study, images were labelled for presence of osteoarthritis in the knee, presence of an old fracture, implants and possible implant complications, tumors and other pathologies. Images were also labelled for fractures in fibula, the diaphyseal femur or diaphyseal tibia. Fractures to the proximal femur and distal tibia are included in the data marked as simply femur fracture or tibia fracture.

| **S1 Table: The base distribution between anatomies. The % is the percentage within that category. The right arrow (→) are for qualifications.** | | | | | | | | | | | | | | | | | |
| --- | --- | --- | --- | --- | --- | --- | --- | --- | --- | --- | --- | --- | --- | --- | --- | --- | --- |
|  | **Train** | | | | | | | |  | **Test** | | | | | | | |
|  | **Fracture** | |  | **Maybe** | |  | **None** | |  | **Fracture** | |  | **Maybe** | |  | **None** | |
|  | **n** | **(%)** |  | **n** | **(%)** |  | **n** | **(%)** |  | **n** | **(%)** |  | **n** | **(%)** |  | **n** | **(%)** |
| **Fracture** | | | | | | | | | | | | | | | | | |
| Fracture | 1,375 | (24) |  | 286 | (5) |  | 4,039 | (71) |  | 146 | (24) |  | 7 | (1) |  | 447 | (74) |
| Femur | 181 | (3) |  | 31 | (1) |  | 5,488 | (96) |  | 20 | (3) |  | 0 | (0) |  | 580 | (97) |
| Femur diaphyseal | 42 | (1) |  | 0 | (0) |  | 5,658 | (99) |  | 7 | (1) |  | 0 | (0) |  | 593 | (99) |
| Femur distal | 119 | (2) |  | 28 | (0) |  | 5,553 | (97) |  | 12 | (2) |  | 0 | (0) |  | 588 | (98) |
| Fibula | 211 | (4) |  | 14 | (0) |  | 5,475 | (96) |  | 32 | (5) |  | 0 | (0) |  | 568 | (95) |
| Hardware fracture |  |  |  |  |  |  |  |  |  | 3 | (0) |  | 0 | (0) |  | 597 | (100) |
| Old fracture | 303 | (5) |  | 0 | (0) |  | 5,397 | (95) |  | 17 | (3) |  | 0 | (0) |  | 583 | (97) |
| Patella | 457 | (8) |  | 68 | (1) |  | 5,175 | (91) |  | 39 | (6) |  | 1 | (0) |  | 560 | (93) |
| Pathological fracture | 3 | (0) |  | 0 | (0) |  | 5,697 | (100) |  | 4 | (1) |  | 0 | (0) |  | 596 | (99) |
| Peri-implant fracture | 12 | (0) |  | 0 | (0) |  | 5,688 | (100) |  | 3 | (0) |  | 0 | (0) |  | 597 | (100) |
| Tibia | 615 | (11) |  | 85 | (1) |  | 5,000 | (88) |  | 71 | (12) |  | 5 | (1) |  | 524 | (87) |
| Tibia diaphyseal | 55 | (1) |  | 0 | (0) |  | 5,645 | (99) |  | 8 | (1) |  | 0 | (0) |  | 592 | (99) |
| Tibia proximal | 543 | (10) |  | 78 | (1) |  | 5,079 | (89) |  | 63 | (10) |  | 5 | (1) |  | 532 | (89) |
| **Distal femur** | | | | | | | | | | | | | | | | | |
| A | | | | | | | | | | | | | | | | | |
| Base | 68 | (1) |  | 24 | (0) |  | 5,608 | (98) |  | 5 | (1) |  | 0 | (0) |  | 595 | (99) |
| 1 | 31 | (1) |  | 23 | (0) |  | 5,646 | (99) |  | 1 | (0) |  | 0 | (0) |  | 599 | (100) |
| …1 | 10 | (0) |  | 7 | (0) |  | 5,683 | (100) |  |  |  |  |  |  |  |  |  |
| …2 | 21 | (0) |  | 16 | (0) |  | 5,663 | (99) |  | 1 | (0) |  | 0 | (0) |  | 599 | (100) |
| 2 | 31 | (1) |  | 1 | (0) |  | 5,668 | (99) |  | 4 | (1) |  | 0 | (0) |  | 596 | (99) |
| …1 | 13 | (0) |  | 0 | (0) |  | 5,687 | (100) |  | 1 | (0) |  | 0 | (0) |  | 599 | (100) |
| …2 | 7 | (0) |  | 0 | (0) |  | 5,693 | (100) |  | 3 | (0) |  | 0 | (0) |  | 597 | (100) |
| …3 | 11 | (0) |  | 1 | (0) |  | 5,688 | (100) |  |  |  |  |  |  |  |  |  |
| 3 | 5 | (0) |  | 0 | (0) |  | 5,695 | (100) |  |  |  |  |  |  |  |  |  |
| …1 | 1 | (0) |  | 0 | (0) |  | 2,521 | (44) |  | 0 | (0) |  | 0 | (0) |  | 0 | (0) |
| …2 | 1 | (0) |  | 0 | (0) |  | 2,521 | (44) |  | 0 | (0) |  | 0 | (0) |  | 0 | (0) |
| …3 | 3 | (0) |  | 0 | (0) |  | 5,697 | (100) |  |  |  |  |  |  |  |  |  |
| B | | | | | | | | | | | | | | | | | |
| Base | 31 | (1) |  | 4 | (0) |  | 5,665 | (99) |  | 4 | (1) |  | 0 | (0) |  | 596 | (99) |
| 1 | 15 | (0) |  | 2 | (0) |  | 5,683 | (100) |  | 2 | (0) |  | 0 | (0) |  | 598 | (100) |
| …1 | 9 | (0) |  | 1 | (0) |  | 5,690 | (100) |  | 2 | (0) |  | 0 | (0) |  | 598 | (100) |
| …2 | 3 | (0) |  | 0 | (0) |  | 2,519 | (44) |  | 0 | (0) |  | 0 | (0) |  | 0 | (0) |
| …3 | 0 | (0) |  | 0 | (0) |  | 5,700 | (100) |  | 0 | (0) |  | 0 | (0) |  | 0 | (0) |
| 2 | 11 | (0) |  | 0 | (0) |  | 5,689 | (100) |  | 2 | (0) |  | 0 | (0) |  | 598 | (100) |
| …1 | 4 | (0) |  | 0 | (0) |  | 5,696 | (100) |  | 2 | (0) |  | 0 | (0) |  | 598 | (100) |
| …2 | 5 | (0) |  | 0 | (0) |  | 5,695 | (100) |  |  |  |  |  |  |  |  |  |
| …3 | 1 | (0) |  | 0 | (0) |  | 5,699 | (100) |  |  |  |  |  |  |  |  |  |
| 3 | 4 | (0) |  | 2 | (0) |  | 5,694 | (100) |  |  |  |  |  |  |  |  |  |
| …1 | 1 | (0) |  | 2 | (0) |  | 2,519 | (44) |  | 0 | (0) |  | 0 | (0) |  | 0 | (0) |
| …2 | 2 | (0) |  | 0 | (0) |  | 5,698 | (100) |  |  |  |  |  |  |  |  |  |
| …3 | 1 | (0) |  | 0 | (0) |  | 3,177 | (56) |  | 0 | (0) |  | 0 | (0) |  | 0 | (0) |
| C | | | | | | | | | | | | | | | | | |
| Base | 20 | (0) |  | 0 | (0) |  | 5,680 | (100) |  | 3 | (0) |  | 0 | (0) |  | 597 | (100) |
| 1 | 7 | (0) |  | 0 | (0) |  | 5,693 | (100) |  | 2 | (0) |  | 0 | (0) |  | 598 | (100) |
| …1 | 5 | (0) |  | 0 | (0) |  | 5,695 | (100) |  | 2 | (0) |  | 0 | (0) |  | 598 | (100) |
| …3 | 0 | (0) |  | 0 | (0) |  | 0 | (0) |  | 0 | (0) |  | 0 | (0) |  | 0 | (0) |
| 2 | 9 | (0) |  | 0 | (0) |  | 5,691 | (100) |  | 1 | (0) |  | 0 | (0) |  | 599 | (100) |
| …1 | 1 | (0) |  | 0 | (0) |  | 5,699 | (100) |  | 1 | (0) |  | 0 | (0) |  | 599 | (100) |
| …2 | 2 | (0) |  | 0 | (0) |  | 5,698 | (100) |  |  |  |  |  |  |  |  |  |
| …3 | 5 | (0) |  | 0 | (0) |  | 5,695 | (100) |  |  |  |  |  |  |  |  |  |
| 3 | 3 | (0) |  | 0 | (0) |  | 2,519 | (44) |  | 0 | (0) |  | 0 | (0) |  | 0 | (0) |
| …3 | 2 | (0) |  | 0 | (0) |  | 2,520 | (44) |  | 0 | (0) |  | 0 | (0) |  | 0 | (0) |
| Lateral | 2 | (0) |  | 0 | (0) |  | 2,520 | (44) |  | 1 | (0) |  | 0 | (0) |  | 599 | (100) |
| Medial | 1 | (0) |  | 0 | (0) |  | 2,521 | (44) |  | 0 | (0) |  | 0 | (0) |  | 0 | (0) |
| **Patella** | | | | | | | | | | | | | | | | | |
| A | | | | | | | | | | | | | | | | | |
| Base | 110 | (2) |  | 45 | (1) |  | 5,545 | (97) |  | 5 | (1) |  | 0 | (0) |  | 595 | (99) |
| 1 | 108 | (2) |  | 43 | (1) |  | 5,549 | (97) |  | 5 | (1) |  | 0 | (0) |  | 595 | (99) |
| 1a | 12 | (0) |  | 10 | (0) |  | 5,678 | (100) |  | 2 | (0) |  | 0 | (0) |  | 598 | (100) |
| 1b | 30 | (1) |  | 4 | (0) |  | 5,666 | (99) |  | 1 | (0) |  | 0 | (0) |  | 599 | (100) |
| 1c | 18 | (0) |  | 7 | (0) |  | 5,675 | (100) |  | 1 | (0) |  | 0 | (0) |  | 599 | (100) |
| 1d | 46 | (1) |  | 18 | (0) |  | 5,636 | (99) |  | 1 | (0) |  | 0 | (0) |  | 599 | (100) |
| B | | | | | | | | | | | | | | | | | |
| Base | 98 | (2) |  | 14 | (0) |  | 5,588 | (98) |  | 5 | (1) |  | 1 | (0) |  | 594 | (99) |
| 1 | 71 | (1) |  | 9 | (0) |  | 5,620 | (99) |  | 5 | (1) |  | 1 | (0) |  | 594 | (99) |
| …1 | 35 | (1) |  | 3 | (0) |  | 5,662 | (99) |  | 2 | (0) |  | 1 | (0) |  | 597 | (100) |
| …2 | 27 | (0) |  | 2 | (0) |  | 5,671 | (99) |  | 3 | (0) |  | 0 | (0) |  | 597 | (100) |
| 2 | 23 | (0) |  | 4 | (0) |  | 5,673 | (100) |  |  |  |  |  |  |  |  |  |
| …1 | 16 | (0) |  | 2 | (0) |  | 5,682 | (100) |  |  |  |  |  |  |  |  |  |
| …2 | 3 | (0) |  | 1 | (0) |  | 5,696 | (100) |  |  |  |  |  |  |  |  |  |
| C | | | | | | | | | | | | | | | | | |
| Base | 249 | (4) |  | 8 | (0) |  | 5,443 | (95) |  | 29 | (5) |  | 0 | (0) |  | 571 | (95) |
| 1 | 125 | (2) |  | 4 | (0) |  | 5,571 | (98) |  | 11 | (2) |  | 0 | (0) |  | 589 | (98) |
| …1 | 60 | (1) |  | 3 | (0) |  | 5,637 | (99) |  | 6 | (1) |  | 0 | (0) |  | 594 | (99) |
| …2 | 14 | (0) |  | 1 | (0) |  | 5,685 | (100) |  |  |  |  |  |  |  |  |  |
| …3 | 47 | (1) |  | 0 | (0) |  | 5,653 | (99) |  | 5 | (1) |  | 0 | (0) |  | 595 | (99) |
| 2 | 49 | (1) |  | 1 | (0) |  | 5,650 | (99) |  | 8 | (1) |  | 0 | (0) |  | 592 | (99) |
| 3 | 74 | (1) |  | 2 | (0) |  | 5,624 | (99) |  | 10 | (2) |  | 0 | (0) |  | 590 | (98) |
| Dislocated | 90 | (2) |  | 5 | (0) |  | 5,605 | (98) |  | 21 | (4) |  | 0 | (0) |  | 579 | (96) |
| **Proximal tibia** | | | | | | | | | | | | | | | | | |
| A | | | | | | | | | | | | | | | | | |
| Base | 113 | (2) |  | 43 | (1) |  | 5,544 | (97) |  | 6 | (1) |  | 4 | (1) |  | 590 | (98) |
| 1 | 87 | (2) |  | 41 | (1) |  | 5,572 | (98) |  | 5 | (1) |  | 3 | (0) |  | 592 | (99) |
| …1 | 43 | (1) |  | 14 | (0) |  | 5,643 | (99) |  | 0 | (0) |  | 3 | (0) |  | 597 | (100) |
| …2 | 8 | (0) |  | 4 | (0) |  | 5,688 | (100) |  |  |  |  |  |  |  |  |  |
| …3 | 36 | (1) |  | 20 | (0) |  | 5,644 | (99) |  | 5 | (1) |  | 0 | (0) |  | 595 | (99) |
| …→a | 14 | (0) |  | 4 | (0) |  | 5,682 | (100) |  | 3 | (0) |  | 0 | (0) |  | 597 | (100) |
| …→p | 6 | (0) |  | 3 | (0) |  | 5,691 | (100) |  | 1 | (0) |  | 0 | (0) |  | 599 | (100) |
| 2 | 24 | (0) |  | 0 | (0) |  | 5,676 | (100) |  | 0 | (0) |  | 1 | (0) |  | 599 | (100) |
| …1 | 1 | (0) |  | 0 | (0) |  | 2,521 | (44) |  | 0 | (0) |  | 0 | (0) |  | 0 | (0) |
| …2 | 9 | (0) |  | 0 | (0) |  | 5,691 | (100) |  |  |  |  |  |  |  |  |  |
| …3 | 13 | (0) |  | 0 | (0) |  | 5,687 | (100) |  | 0 | (0) |  | 1 | (0) |  | 599 | (100) |
| 3 | 2 | (0) |  | 0 | (0) |  | 5,698 | (100) |  | 1 | (0) |  | 0 | (0) |  | 599 | (100) |
| …1 |  |  |  |  |  |  |  |  |  | 1 | (0) |  | 0 | (0) |  | 599 | (100) |
| …2 | 2 | (0) |  | 0 | (0) |  | 5,698 | (100) |  |  |  |  |  |  |  |  |  |
| …3 | 0 | (0) |  | 0 | (0) |  | 5,700 | (100) |  | 0 | (0) |  | 0 | (0) |  | 0 | (0) |
| Dislocated | 11 | (0) |  | 0 | (0) |  | 5,689 | (100) |  | 3 | (0) |  | 0 | (0) |  | 597 | (100) |
| B | | | | | | | | | | | | | | | | | |
| Base | 373 | (7) |  | 35 | (1) |  | 5,292 | (93) |  | 46 | (8) |  | 1 | (0) |  | 553 | (92) |
| 1 | 81 | (1) |  | 9 | (0) |  | 5,610 | (98) |  | 11 | (2) |  | 0 | (0) |  | 589 | (98) |
| …1 | 34 | (1) |  | 8 | (0) |  | 5,658 | (99) |  | 6 | (1) |  | 0 | (0) |  | 594 | (99) |
| …2 | 16 | (0) |  | 1 | (0) |  | 5,683 | (100) |  | 2 | (0) |  | 0 | (0) |  | 598 | (100) |
| …3 | 28 | (0) |  | 0 | (0) |  | 5,672 | (100) |  | 3 | (0) |  | 0 | (0) |  | 597 | (100) |
| 2 | 169 | (3) |  | 22 | (0) |  | 5,509 | (97) |  | 9 | (2) |  | 1 | (0) |  | 590 | (98) |
| …1 | 149 | (3) |  | 16 | (0) |  | 5,535 | (97) |  | 6 | (1) |  | 0 | (0) |  | 594 | (99) |
| …2 | 20 | (0) |  | 5 | (0) |  | 5,675 | (100) |  | 3 | (0) |  | 1 | (0) |  | 596 | (99) |
| 3 | 118 | (2) |  | 1 | (0) |  | 5,581 | (98) |  | 26 | (4) |  | 0 | (0) |  | 574 | (96) |
| …1 | 56 | (1) |  | 1 | (0) |  | 5,643 | (99) |  | 12 | (2) |  | 0 | (0) |  | 588 | (98) |
| …2 | 7 | (0) |  | 0 | (0) |  | 5,693 | (100) |  | 0 | (0) |  | 0 | (0) |  | 0 | (0) |
| …3 | 56 | (1) |  | 0 | (0) |  | 5,644 | (99) |  | 14 | (2) |  | 0 | (0) |  | 586 | (98) |
| →lx | 31 | (1) |  | 1 | (0) |  | 2,490 | (44) |  | 5 | (1) |  | 0 | (0) |  | 595 | (99) |
| →mx | 5 | (0) |  | 0 | (0) |  | 2,517 | (44) |  | 1 | (0) |  | 1 | (0) |  | 598 | (100) |
| →t | 29 | (1) |  | 2 | (0) |  | 2,491 | (44) |  | 7 | (1) |  | 0 | (0) |  | 593 | (99) |
| →u | 35 | (1) |  | 1 | (0) |  | 2,486 | (44) |  | 6 | (1) |  | 0 | (0) |  | 594 | (99) |
| →v | 7 | (0) |  | 0 | (0) |  | 2,515 | (44) |  | 1 | (0) |  | 0 | (0) |  | 599 | (100) |
| →w | 1 | (0) |  | 2 | (0) |  | 2,519 | (44) |  | 0 | (0) |  | 0 | (0) |  | 0 | (0) |
| C | | | | | | | | | | | | | | | | | |
| Base | 57 | (1) |  | 0 | (0) |  | 5,643 | (99) |  | 11 | (2) |  | 0 | (0) |  | 589 | (98) |
| 1 | 12 | (0) |  | 0 | (0) |  | 5,688 | (100) |  | 2 | (0) |  | 0 | (0) |  | 598 | (100) |
| …1 | 8 | (0) |  | 0 | (0) |  | 5,692 | (100) |  | 2 | (0) |  | 0 | (0) |  | 598 | (100) |
| …2 | 3 | (0) |  | 0 | (0) |  | 5,697 | (100) |  |  |  |  |  |  |  |  |  |
| 2 | 11 | (0) |  | 0 | (0) |  | 5,689 | (100) |  | 4 | (1) |  | 0 | (0) |  | 596 | (99) |
| …1 | 3 | (0) |  | 0 | (0) |  | 5,697 | (100) |  | 1 | (0) |  | 0 | (0) |  | 599 | (100) |
| …2 | 6 | (0) |  | 0 | (0) |  | 5,694 | (100) |  | 2 | (0) |  | 0 | (0) |  | 598 | (100) |
| …3 | 2 | (0) |  | 0 | (0) |  | 5,698 | (100) |  | 1 | (0) |  | 0 | (0) |  | 599 | (100) |
| 3 | 31 | (1) |  | 0 | (0) |  | 5,669 | (99) |  | 5 | (1) |  | 0 | (0) |  | 595 | (99) |
| …1 | 22 | (0) |  | 0 | (0) |  | 5,678 | (100) |  | 4 | (1) |  | 0 | (0) |  | 596 | (99) |
| …2 | 3 | (0) |  | 0 | (0) |  | 5,697 | (100) |  | 0 | (0) |  | 0 | (0) |  | 0 | (0) |
| …3 | 6 | (0) |  | 0 | (0) |  | 5,694 | (100) |  | 1 | (0) |  | 0 | (0) |  | 599 | (100) |
| Dislocated | 49 | (1) |  | 0 | (0) |  | 2,473 | (43) |  | 29 | (5) |  | 0 | (0) |  | 571 | (95) |
| Into diaphysis | 8 | (0) |  | 0 | (0) |  | 2,514 | (44) |  | 5 | (1) |  | 0 | (0) |  | 595 | (99) |
| Lateral | 97 | (2) |  | 11 | (0) |  | 5,592 | (98) |  | 12 | (2) |  | 2 | (0) |  | 586 | (98) |
| Medial | 37 | (1) |  | 2 | (0) |  | 5,661 | (99) |  | 9 | (2) |  | 1 | (0) |  | 590 | (98) |

# Cohen Kappa values

**S2 Table: Inter-rater reliability between MG and AE based on Cohen’s kappa values.**

| **Inter-rater reliability** | | | | | | |
| --- | --- | --- | --- | --- | --- | --- |
|  |  |  | **% identified in the category** | |  |  |
|  | **Cohen’s kappa** |  | **Reviewer 1** | **Reviewer 2** |  | **Agreement** |
| **Fracture** | 1.00 |  | 39 | 39 |  | 100 |
| **Proximal tibia** | | | | | | |
| Base | 0.95 |  | 17 | 17 |  | 98 |
| A | 0.78 |  | 3 | 3 |  | 99 |
| B | 0.90 |  | 11 | 11 |  | 98 |
| C | 0.79 |  | 3 | 4 |  | 99 |
| A1 | 0.89 |  | 3 | 2 |  | 99 |
| A2 | 0.00 |  | 0 | 2 |  | 99 |
| B1 | 0.23 |  | 2 | 2 |  | 97 |
| B11 | 0.44 |  | 1 | 2 |  | 99 |
| B2 | 0.36 |  | 3 | 2 |  | 97 |
| B21 | 0.40 |  | 2 | 1 |  | 98 |
| B3 | 0.67 |  | 6 | 8 |  | 96 |
| B31 | 0.66 |  | 3 | 4 |  | 98 |
| B33 | 0.60 |  | 3 | 3 |  | 98 |
| B -> lx | 0.17 |  | 2 | 1 |  | 98 |
| B -> t | 0.36 |  | 2 | 3 |  | 97 |
| B -> u | 0.60 |  | 2 | 1 |  | 99 |
| C1 | 0.00 |  | 1 | 0 |  | 99 |
| C2 | 0.28 |  | 1 | 1 |  | 99 |
| C3 | 0.13 |  | 1 | 3 |  | 97 |
| B2 or B3 lateral | 0.83 |  | 5 | 4 |  | 98 |
| B2 or B3 medial | 0.39 |  | 1 | 2 |  | 98 |
| C2 or C3 fragmented | 0.40 |  | 1 | 1 |  | 99 |
| Dislocated | 0.71 |  | 6 | 6 |  | 97 |
| Lateral | 0.40 |  | 3 | 3 |  | 96 |
| Medial | 0.41 |  | 3 | 2 |  | 97 |
| **Patella** | | | | | | |
| Base | 0.99 |  | 10 | 10 |  | 100 |
| A | 0.59 |  | 1 | 1 |  | 99 |
| B | 0.44 |  | 2 | 1 |  | 99 |
| C | 0.93 |  | 7 | 8 |  | 99 |
| A1 | 0.59 |  | 1 | 1 |  | 99 |
| B1 | 0.44 |  | 2 | 1 |  | 99 |
| B11 | 0.40 |  | 1 | 1 |  | 99 |
| B12 | 0.00 |  | 3 | 0 |  | 99 |
| C1 | 0.70 |  | 2 | 2 |  | 99 |
| C11 | 0.39 |  | 1 | 2 |  | 98 |
| C13 | 0.33 |  | 1 | 0 |  | 99 |
| C2 | 0.30 |  | 2 | 3 |  | 97 |
| C3 | 0.66 |  | 3 | 3 |  | 98 |
| Dislocated | 0.88 |  | 6 | 5 |  | 99 |
| **Distal femur** | | | | | | |
| Base | 0.87 |  | 3 | 3 |  | 99 |
| A | 0.72 |  | 1 | 2 |  | 99 |
| B | 0.86 |  | 1 | 1 |  | 100 |
| C | 0.80 |  | 1 | 1 |  | 100 |
| B11 or C11 | 0.66 |  | 1 | 1 |  | 99 |

## Reconciliation through consensus

## Out of the 600 exams in the test-set, 71 cases had a mismatch for either general fracture or one of the AO/OTA categories that required reconciliation. 41 cases had a mismatch for proximal tibia, 21 for patella and 4 for the distal femur. For type, group and subgroup the corresponding numbers are 16, 48 and 31. The platform for labeling allows for not labeling as far as possible, i.e., if a user feels that they are unable to put a proper class on that category they can skip it.

# References

1. Meinberg E, Agel J, Roberts C, al. e. Fracture and Dislocation Classification Compendium - 2018. Journal of Orthopaedic Trauma. 2018;32(January):170.
