# Supplementary material for: Artificial intelligence for the classification of fractures around the knee in adults according to the 2018 AO/OTA classification system
Source: PLoS One. 2021 Apr 1;16(4):e0248809. doi: 10.1371/journal.pone.0248809 (PMC8016258; doi:10.1371/journal.pone.0248809)
Supplement: Attachment — Submitted filename: 2nd rebuttal.docx [file pone.0248809.s003.docx]

# Response to reviewers – 2:nd rebuttal

**Reviewer #1: Thank you for your thorough responses. I have a few follow-up queries**

**On line 65 perhaps clarify that you did not look at all plain radiographs but rather those around the knee joint.**

Response: Thank you for noting this.
Action: We have clarified this in the text.

**Consider re-writing the next paragraph so it is clear who excluded images for quality or open physes etc.**

Response: We have tried to clarify this further. The platform does not allow regular users to fully remove images, they were initially tagged for removal and then reviewed a second time MG before exclusion.
Action: We have tried to make this clearer in the paragraph.

**Line 102 - there are three sets of initials but only 2 orthopedic surgeons in your text**

Response: Our platform allows for choosing the number of reviewers per exam. I reviewed the initial batch of 395 images but then I had some other tasks that I had to prioritize while OS had time to go through some additional images so that we got a decent amount of test cases. EA reviewed all 600. As all of us are experienced orthopedic surgeons and familiar with the classification, this approach was chosen for convenience.
Action: None

**I am sorry if I missed this but did you report the agreement between your human raters -- how far down into the codes did they go - how many cases needed reconciliation**

Response: In total 71 cases had a mismatch for either general fracture or one of the AO/OTA categories that required reconciliation. 41 cases had a mismatch for proximal tibia, 21 for patella and 4 for the distal femur. For type, group and subgroup the corresponding numbers are 16, 48 and 31. The platform for labeling allows for not labeling as far as possible, i.e., if a user feels that they are unable to put a proper class on that category they can skip it.
Action: We have added the total number of reconciliations to the manuscript, the details are in the supplement.

**You have very few distal femur training cases - is this number adequate?**

Response: Yes, it is correct. We tried to identify more distal femoral fractures as we expanded the dataset so that we could get a more complete representation of all the classes. Our clinical gut feeling is that these have increased at our institution recent years, many that are peri-implant fractures, and this could be a side-effect from the source data spanning a historical period (2002-2016).
Action: None

**Were all the A cases evaluated for being A so in the patella you actually have 12 A's , 10 A1 etc**

Response: The A-category in patella is somewhat of a special case. It only has A1 and there are only qualifiers. The correct is that there are 5 A fractures that all belong to the A1 class where 2 also had the qualifier “a”. As we set the threshold to 2 examples in the evaluation we could not include the other 3:s subcategories as they were evenly spread over the categories. Presenting the AO-classification is challenging and we chose this method as we have found it to correspond well to how it works from a practical perspective, when we work with the network and we disagree with some class we want the network to provide alternative interpretations. It is undesirable to only receive a single prediction.
Action: We have clarified in the text to better explain this presentation.

**Thank you for these clarifications on your interesting work**

**Reviewer #3: The revision has improved this paper in several aspects. However, the method (neural network) is still not sufficiently described in my opinion. The argument that the code will be published on acceptance does not appeal to me - the reader should not need to dig through your code to understand your method.**

**I would strongly recommend moving most of the neural network description from the supplement into the main body of the manuscript, and extending this description. The reader should be able to reimplement the method based on the description in the paper, but there are currently too many details missing.**

**The “Neural network setup” section needs to contain more details about the whole setup, most importantly how the individual regularization techniques were used together (the reference for using auto-encoders for regularization [21] also refers to a paper about Stochastic Weight Averaging, this seems to be a mistake).**

Response: This is a valid point and we agree that readers should not have to dig into the code/supplement for finding the most important details. You are also right about that references got a little mixed up. Except for noise each regularizing strategy was used in isolation between each session.
Action: We have moved the supplement into the main manuscript and tried to clarify it more. We have also added more columns to the regularizers so that it is more explicitly clear that the regularizers were run in isolation of each other.

**The outputs of the network appear to be sigmoid units, but the table in the supplement lists only 4 output units - it is still not clear to me how these are translated into the different categories. Or is there another layer with the final output units? With sigmoid outputs, two classifications that exclude each other could both have the same probability (e.g. 1.0) - what would be the final classification in such a case?**

Response: This is correct, each output was converted using a sigmoid. In our labeling application we use a softmax for categories if they are mutually exclusive if the sum of the sigmoid exceeds 1, e.g. the B1, B2, and B3 are mutually exclusive and we get the probabilities 0.6, 0.9, 0.1 we would convert them into 0.375, 0.5625 and 0.0625. If the model doesn’t find that any of the categories are a good match, we leave the probabilities without intervention, e.g. 0.1, 0.3, 0.1 sums to 0.5 and a softmax would incorrectly boost the B2 category and under some circumstances it may happen that none of the options is a good fit where I as a clinician would not have chosen any of the categories. This is more though of a clinical application problem and is part of the network calibration procedure. Our intention of the paper is to show the ability to identify each category and separate it from the remainder, therefore we use AUC as our core measure for each of the classes.

The major issue with the classification tree is that visually similar fractures can belong in entirely different branches and it becomes tricky to properly choose group, e.g. proximal tibia B21 and B31 and more similar than their neighboring classes. In our labeling application we let the network prediction select categories if the prediction is over 60% and this usually corresponds to the correct group and usually the network can correctly set the type and group category where the user must choose at the subgroup category.
Action: We added a short description on the labeling platform in the methods section.

**“The test set consisted of 600 cases, which were classified by two senior orthopedic surgeons, MG, OS and EA, working independently.” - this seems to list the initials of three observers?**

Response: See above question from your fellow reviewer

**The data availability statement has been considerably improved, now the test set will be released. The authors could consider also uploading their evaluation pipeline and their results to a platform like** [**grand-challenge.org**](http://grand-challenge.org/) **where other researchers can then later upload their own predictions for comparison.**

Response: Thank you for the suggestion. We have prepared a data sharing agreement with AIDA for open data sharing, <https://aida-dataset-register.github.io/sharing/>. Our lawyers at KI found this approach to be an ideal approach that gives us piece of mind when it comes to GDPR and other regulations that we must respect. The data is uploaded and will be published as soon as PLOS One provides us with a DOI that we can link to.
